# Analysis of risk factors related to acute exacerbation in patients with chronic pancreatitis: a retrospective study of 313 cases from a single center in China

**DOI:** 10.1186/s12876-024-03528-w

**Published:** 2024-11-27

**Authors:** Jiaming Liu, Cong Wang, Zhen Chen, Qili Dai, Jingrui Bai, Yun‑Feng Cui

**Affiliations:** 1 Tianjin Medical University, Tianjin, 300070 China; 2 Department of Hepatobiliary and Pancreatic Surgery, Department of Surgery, Nankai Clinical School of Medicine, Tianjin Nankai Hospital, Tianjin Medical University, 6 Changjiang Road, Nankai District, Tianjin, 300100 China

**Keywords:** Chronic pancreatitis, Acute on chronic pancreatitis, Recurrent acute pancreatitis, Age, Steatorrhea

## Abstract

**Background:**

Acute on chronic pancreatitis(ACP) is a common cause of treatment in patients with chronic pancreatitis(CP). However, as far as we know, research on ACP has been few, and the quality may vary. This study intended to explore the risk factors related to acute exacerbation in patients with chronic pancreatitis.

**Methods:**

313 patients with CP were analyzed based on clinical data from 2014 to 2023 and categorized into ACP and non-ACP groups. Their data, assessed across eleven parameters, were used to study risk variables associated with acute exacerbation in patients with chronic pancreatitis.

**Results:**

Of the 313 eligible patients, 163(52.1%) were ACP. Age > 50 years old (*P* = 0.049, *OR* = 0.614, 95%*CI*: 0.378–0.998), recurrent acute pancreatitis(RAP) history (*P* = 0.000, *OR* = 3.284, 95%*CI*: 1.972–5.467) and steatorrhea (*P* = 0.013, *OR* = 0.189, 95%*CI*: 0.051–0.704) were related factors for ACP.

**Conclusion:**

The history of RAP was an independent risk factor for ACP. Age and steatosis were protective of the prevalence of ACP.

## Background

CP, or chronic progressive pancreatic inflammation, results from a combination of environmental, genetic, and other factors, often leading to irreversible damage to the morphology and function of the pancreas [1]. Over time, fibrous tissue progressively replaces pancreatic tissue, leading to failure in both endocrine and exocrine functions, which can present as diabetes and steatorrhea. Additionally, some CP patients may develop acute pancreatic inflammation, known as ACP, which commonly complicates the course of CP [2]. However, research related to ACP has been limited by the absence of widely accepted definitions and diagnostic criteria, resulting in variable quality studies. To address this issue, Tiago et al. recently released a comprehensive statement on ACP in collaboration with several renowned pancreatic centers worldwide, providing clear definitions, diagnostic criteria, and distinguishing features of ACP compared to CP [3, 4]. Building upon this statement, the present study summarizes the clinical characteristics of 163 patients diagnosed with ACP in our hospital and discusses the risk factors associated with ACP admission.

## Methods

### Patient identification and selection

From June 2014 to January 2023, patients diagnosed with CP were assessed for eligibility upon admission to the Emergency and Hepatopancreatobiliary Surgery Departments at Nankai Hospital (Tianjin, China). Demographic and clinical data were collected from patients upon admission. All patients were diagnosed as CP as per Asia Pacific consensus [5], while ACP patients were diagnosed based on the new position statement on acute on chronic pancreatitis issued in 2023 [3].

Patients that met the following criteria were not included: (1) Under 18 years old. (2) Patients with a diagnosis of autoimmune pancreatitis. (3) Patients who are suspected or have been diagnosed with groove pancreatitis. (4) Patients with pancreatic malignant tumors include pancreatic cancer, ampullary carcinoma, and pancreatic intraductal papillary mucinous tumor. (5) Patients with suspected pancreatic cancer, pancreatic intraductal papillary mucinous tumor, pancreatic serous cystadenoma, mucinous cystadenoma, pancreatic neuroendocrine tumor, and other pancreatic malignant or potentially malignant tumors showing pancreatic calcification, pending confirmation by pathology. (6) Patients with a history of prior pancreatic surgery.

### Data collection

Data about clinical characteristics, including laboratory parameters, imaging records, and disease phases, were recorded. The metrics analyzed in this study included demographic characteristics such as age, gender, risk factors, and clinical parameters such as the M-ANNHEIM severity score and M-ANNHEIM staging [6]. All laboratory results were obtained from the Central Laboratory of Nankai Hospital following standard protocols.

### Statistics

Data analysis was conducted using SPSS 25.0 software (IBM SPSS Statistics; IBM Corporation; Armonk, NY). The distributions of quantitative variables were tested, with normally and non-normally distributed variables presented as the median (interquartile range), respectively. Continuous variables were compared between groups using unpaired *t*-tests and paired *t*-tests within each group. Categorical variables were compared using the *Chi-square* test. For small samples, analysis of variance and *Fisher’s exact* test were employed to analyze continuous and categorical variables, respectively. Statistical significance was defined as *P* < 0.05. Several series of univariate logistic regression analyses were conducted to identify risk factors for ACP involving the 11 indices (age, gender, BMI, risk factor, clinical manifestation, complication, pancreatic dilatation, pancreatic stricture, pancreatic atrophy, M-ANNHEIM severity score, M-ANNHEIM staging). The stepwise method further tested Variables showing statistical significance in multiple logistic regression analyses.

### Results

There were 313 individuals in the trial, and 163 were diagnosed with ACP. Table 1 displays the ACP and non-ACP groups’ demographic data and clinical characteristics. Patients in the non-ACP group had a higher average age than those in the ACP group (*p* < 0.05). Moreover, the non-ACP group exhibited a higher proportion of patients with weight loss, steatorrhea, pancreatic duct dilation, and pancreatic atrophy compared to the ACP group (*P* < 0.05). Conversely, the ACP group showed a higher proportion of patients experiencing abdominal pain, nausea, and vomiting compared to the non-ACP group (*P* < 0.05). Laboratory test results for patients in both the ACP and non-ACP groups are presented in Table 2. Patients in the ACP group exhibited significantly higher blood and urinary amylase levels than those in the non-ACP group. Only 54 patients in the ACP group had serum amylase, lipase, or both levels exceeding three times the upper limit of normal.

**Table 1:** Demographic data and clinical characteristics of the patients with CP

Characteristic	Total (*n* = 313)	ACP (*n* = 163)	Non-ACP (*n* = 150)	*P* value
Age, years	52.4 ± 13.1	49.9 ± 13.4	55.1 ± 12.4	0.000404
Gender, M	263	142	121	0.120
BMI, kg/m2	21.7 ± 3.4	21.6 ± 3.4	21.7 ± 3.4	0.832
Risk factor				
Smoking	164	89	75	0.415
Alcohol	203	111	92	0.210
RAP history	110	79	31	0.000
Gallstone	41	22	19	0.828
Fatter liver	57	32	25	0.497
Hyperlipidemia	28	12	16	0.306
Clinical manifestation				
Abdominal pain	262	158	104	3.9755E-11
Radiation pain	110	62	48	0.264
Nausea and vomiting	81	52	29	0.011
Jaundice	23	12	11	0.992
Weight loss	60	23	37	0.018
Complication				
Diabetes	81	37	44	0.181
Steatorrhea	18	3	15	0.002
PPC	88	47	41	0.768
CBDS	51	21	30	0.089
Thrombosis	9	7	2	0.220
Pancreatic duct dilatation	227	109	118	0.020
Pancreatic duct stricture	14	8	6	0.698
Pancreatic atrophy	43	14	29	0.006
M-ANNHEIM severity score	8.8 ± 2.6	9.0 ± 2.2	8.6 ± 3.0	0.162
M-ANNHEIM staging				0.003
I	215	123	92	
II	85	38	47	
III	6	2	4	
IV	7	0	7	

CP chronic pancreatitis, ACP acute on chronic pancreatitis, RAP recurrent acute pancreatitis, M male, F female, BMI body mass index, PPC pancreatic pseudocyst, CBDS common bile duct stricture

**Table 2:** Laboratory tests of the patients with CP

laboratory tests	Total (*n* = 313)	ACP (*n* = 163)	Non-ACP (*n* = 150)	*P* value
Serum amylase	96(47–176)	116(41–176)	74(57–176)	0.010
Urinary amylase	476(136–957)	556(94–918)	451(187–556)	0.002
AFP	5.5(3.3–7.8)	5.4(3.7–7.6)	5.5(3.8–7.7)	0.962
CEA	4.9(2.6–7.7)	5.2(2.4–8.4)	3.6(2.8–6.8)	0.159
CA19-9	22.5(13.3–30.8)	21.4(13.3–30.2)	23.4(13.8–30.8)	0.721

CP chronic pancreatitis, ACP acute on chronic pancreatitis

Table 3 displays the univariate regression results for ACP in CP. Significant differences were observed in age, history of RAP, weight loss, steatorrhea, diabetes, pancreatic duct dilation, and pancreatic atrophy between the two groups. These significant variables were included in the multiple logistic regression analysis model based on the results of univariate analysis. Table 4 demonstrates that a history of RAP was identified as an independent risk factor for ACP in CP, while age > 50 years and steatorrhea were protective factors against acute exacerbation in CP patients.

**Table 3:** Univariate logistic regression analysis of ACP

Variable	OR	95% C I		*P* value
		Lower	Upper	
Age>50	0.542	0.342	0.858	0.009
RAP history	3.610	2.188	5.956	0.000
Weight loss	0.502	0.282	0.893	0.019
Steatorrhea	0.169	0.048	0.595	0.006
Pancreatic duct dilatation	0.547	0.329	0.911	0.020
Pancreatic atrophy	0.392	0.198	0.775	0.007
M-ANNHEIM staging	0.516	0.348	0.764	0.001

**Table 4:** Independent risk factors in a multivariate logistic regression analysis of ACP

Variable	OR	95% C I		*P* value
		Lower	Upper	
Age>50	0.614	0.378	0.998	0.049
RAP history	3.284	1.972	5.467	0.000
Steatorrhea	0.189	0.051	0.704	0.013

CP chronic pancreatitis, ACP acute on chronic pancreatitis, RAP recurrent acute pancreatitis, M male, F female, BMI body mass index

## Discussion

The lack of a universally agreed-upon definition of ACP has hindered properly determining its incidence. Approximately 12% of patients with acute pancreatitis (AP) admitted to the hospital have pancreatic duct stones or pancreatic calcification, as stated in the literature [7]. Another cohort study found that only 3.5% of 1415 CP patients were diagnosed with ACP [8]. But in this study, out of 313 CP patients, 163 (52.1%)were diagnosed with ACP, among which 60 were admitted to the hospital due to AP. The incidence rate is higher than that in earlier literature, and the author believes that there may be the following reasons. This study was taken up based on the new position statement on ACP issued in 2023 [3]. This includes not only the ACP diagnosis of known CP, but also the ACP diagnosis of unknown CP. In addition, recent literature has shown that the rate of ACP diagnosis during emergency department visits has increased, and the incidence of ACP relative to AP appears to be increasing.This might reflect a true increase in the incidence of ACP or alternatively an increased awareness of ACP, and improved diagnostic accuracy due to the more widespread use of cross-sectional imaging modalities.

Patient data from both the ACP and non-ACP groups were compared and analyzed. The BMI and sex distribution did not show significant variations between the two groups, although the ACP group had a lower average age and M-ANNHEIM stage. The authors suggest this pattern could be due to less pancreatic fibrosis in younger individuals. Previous studies have indicated that pancreatic fibrosis may restrict inflammatory necrosis, and there is also a positive correlation between age and the extent of pancreatic fibrosis [9–11].

Because of the similarities between ACP and AP, previous guidelines recommended utilizing the diagnostic and treatment strategies for acute pancreatitis to address chronic pancreatitis; furthermore, it has been suggested that ACP shares similar risk factors with AP, including drinking and smoking [12]. However, this approach may not be appropriate, as the pathogenesis of the two conditions is not entirely identical [1, 2, 13]. Evidence suggests that alcohol consumption may preferentially induce ACP rather than AP [14]. Additionally, recent studies by Olesen et al. have demonstrated that smoking is associated with fibrosis-related complications but not with ACP [15]. In this study, the authors compared the distribution of risk factors between the two groups. The results indicated that there was no significant difference in the proportions of drinking, smoking, cholelithiasis, fatty liver, and hyperlipidemia between the two groups. However, the proportion of patients with a previous history of RAP in the ACP group was significantly higher than that in the non-ACP group.

The CP sentinel event hypothesis suggests that AP-RAP-CP is a continuum, where all CP patients are anticipated to have a history of RAP before diagnosis [16–18]. However, this is not always the case. Only110 out of 313 (35%) patients in this study had a clear history of RAP before CP diagnosis, a proportion close to the 25% reported by Hori et al. [19, 20]. It is noteworthy that such a substantial proportion of patients do not experience RAP attacks before CP diagnosis. Some scholars propose that CP may develop through mechanisms independent of AP. Endoplasmic reticulum stress is one such mechanism supported by evidence from experiments on mice and other animals [8, 21, 22]. Furthermore, research indicates that drinking and smoking can promote pancreatic acinar cell apoptosis and induce CP by triggering endoplasmic reticulum stress and inhibiting the adaptive unfolded protein response signal pathway [21, 23]. Therefore, CP can manifest not only through RAP but also via other independent mechanisms. The authors speculate that patients with a history of RAP remain susceptible to inflammatory injury even after CP diagnosis, rendering them more prone to acute inflammation than those without prior RAP. Moreover, studies suggest that CP patients with a history of RAP tend to be younger than those without such a history, potentially contributing to their increased likelihood of developing ACP [9].

The typical symptoms and signs of ACP patients often overlap with those of AP patients, with pain, nausea, and vomiting commonly observed. In this study, both groups’ clinical manifestations and complications were compared. The results revealed that the proportion of patients experiencing abdominal pain, nausea, vomiting, and radiation pain was significantly higher in the ACP group compared to the non-ACP group. Conversely, the proportion of patients with weight loss, diarrhea, pancreatic atrophy, and pancreatic duct dilatation was significantly lower in the ACP group compared to the non-ACP group. The authors speculate that the reasons for the lower proportion of weight loss, diarrhea, pancreatic atrophy, and pancreatic duct dilatation in the ACP group may be similar to those for the younger average age and lower M-ANNHEIM stage in the ACP group. The occurrence of ACP is related to the residual function of the pancreas. With the continuous progress of CP and the continuous onset of ACP, pancreatic fibrosis and pancreatic atrophy are aggravated, and pancreatic function is gradually lost. When the secretion of pancreatic lipase falls below 10%, there will be obvious steatosis and weight loss. At this time, CP is in the late stage of the disease, pancreatic atrophy, fibrosis is serious, the number of acinar cells decreased sharply, it is difficult to induce acute inflammation [10, 24].

In the latest statement on ACP, the diagnosis is categorized into previously diagnosed CP and undiagnosed CP [3]. The former resembles AP diagnosis and necessitates abdominal pain in addition to amylase/lipase levels exceeding three times the normal range or imaging evidence of acute pancreatic inflammation. Once CP diagnosis is confirmed for the latter, ACP diagnosis requires only the presence of acute pancreatic inflammation without necessitating clinical manifestations like abdominal pain. Additionally, differential diagnosis of ACP includes distinguishing it from post-ERCP AP, post-pancreatectomy AP, and exacerbated CP pain.

However, the utility of amylase/lipase in diagnosing ACP patients in this study was limited, with only 54(33.1%) ACP patients exhibiting amylase/lipase levels exceeding three times the normal value. This may be caused by the secondary fibroinflammatory process associated with CP and the decreased mass of acinar cells in pancreatic atrophy [25]. In a study of patients with ACP, serum amylase and lipase levels tripled above the normal limit in only 20% and 60% of patients and were within the normal range in 36% and 24% of patients, respectively [2]. Therefore, many scholars believe that low levels of serum trypsin can also be accepted for the diagnosis of ACP, but the threshold has not been determined. Besides amylase and lipase, some other blood markers may also contribute to the diagnosis of ACP. It has been found that urinary trypsinogen can be used to identify pancreatitis in patients with pancreatic insufficiency when amylase or lipase levels are normal [26]. In addition, some studies have found that CA19-9 seems to be elevated in ACP, but CA19-9 is not specific and can be significantly increased in pancreatic cancer, cholangitis, and other diseases. In addition, CA19-9 is not expressed in 10% of the population [27, 28]. In this study, the authors compared AFP, CEA, CA19-9, and other tumor markers between the two groups; the results showed no significant difference. In addition to the above markers, Tan et al. have recently found several metabolites that may be highly expressed in ACP patients, including sage acid and auxin b. However, further large-scale trials are needed to verify them [29]. 

In this study of risk factors related to an acute attack of CP, there were seven univariate differences between groups: age, history of RAP, weight loss, steatorrhea, pancreatic duct dilatation, pancreatic atrophy, and M-ANNHEIM stage. Further, it is included in the multi-factor logistic regression model for analysis. The results suggest that age > 50 years (*P* = 0.049, *OR* = 0.614, 95%*CI*: 0.378–0.998), RAP history (*P* = 0.000, *OR* = 3.284, 95%*CI*: 1.972–5.467) and steatorrhea (*P* = 0.013, *OR* = 0.189, 95%*CI*: 0.051–0.704) are associated factors for ACP. The history of RAP is the independent risk factor for ACP. Age and steatorrhea were negatively correlated with the occurrence of ACP. The page reflects that the ACP population itself has some pancreatic function, so it is of great importance to reduce pancreatic inflammation and fibrosis and protect their residual pancreatic function.

While this study may offer some application value for clinical ACP prevention, it is limited by its nature as a single-center, retrospective study with a small sample size and inherent bias. Thus, further validation through multicenter, large-sample, prospective studies is warranted.

## Conclusion

The history of RAP emerged as an independent risk factor for ACP. Age and steatorrhea exhibited a negative correlation with the occurrence of ACP.

## Data Availability

The data that support the findings of this study are not openly available due to reasons of sensitivity and are available from the corresponding author upon reasonable request.

## Abbreviations

AP: Acute pancreatitis
CP: Chronic pancreatitis
ACP: Acute on chronic pancreatitis
RAP: Recurrent acute pancreatitis
M: Male
F: Female
BMI: Body mass index
PPC: Pancreatic pseudocyst
CBDS: Common bile duct stricture
